# Discovery of a novel genetic susceptibility locus on X chromosome for systemic lupus erythematosus

**DOI:** 10.1186/s13075-015-0857-1

**Published:** 2015-12-03

**Authors:** Zhengwei Zhu, Zhuoyuan Liang, Herty Liany, Chao Yang, Leilei Wen, Zhiming Lin, Yujun Sheng, Yan Lin, Lei Ye, Yuyan Cheng, Yan Chang, Lu Liu, Lulu Yang, Yinjuan Shi, Changbing Shen, Fusheng Zhou, Xiaodong Zheng, Jun Zhu, Bo Liang, Yantao Ding, Yi Zhou, Xianyong Yin, Huayang Tang, Xianbo Zuo, Liangdan Sun, Jin-Xin Bei, Jianjun Liu, Sen Yang, Wanling Yang, Yong Cui, Xuejun Zhang

**Affiliations:** Institute of Dermatology and Department of Dermatology, The First Affiliated Hospital, Anhui Medical University, 81 Meishan Road, Hefei, Anhui 230032 China; Key Laboratory of Dermatology, Anhui Medical University, Ministry of Education, Hefei, Anhui 230032 China; Department of Dermatology, China–Japan Friendship Hospital, East Street Cherry Park, Chaoyang District, Beijing, 100029 China; Department of Nephrology, Jiangmen Central Hospital, Jiangmen, Guangdong 529030 China; Human Genetics, Genome Institute of Singapore, Agency for Science Technology and Research, Singapore, Singapore; Department of Rheumatology, The Third Affiliated Hospital of Sun Yat-Sen University, Guangzhou, Guangdong 510630 China; Saw Swee Hock School of Public Health, National University of Singapore, Singapore, Singapore; Department of Paediatrics and Adolescent Medicine, Queen Mary Hospital, LKS Faculty of Medicine, The University of Hong Kong, 21 Sassoon Road, Hong Kong, China; Centre for Genomic Sciences, LKS Faculty of Medicine, The University of Hong Kong, Hong Kong, China

**Keywords:** Systemic lupus erythematosus, X chromosome, Genetics, Single nucleotide polymorphisms

## Abstract

**Introduction:**

Systemic lupus erythematosus (SLE) is an autoimmune connective tissue disease affecting predominantly females. To discover additional genetic risk variants for SLE on the X chromosome, we performed a follow-up study of our previously published genome-wide association study (GWAS) data set in this study.

**Methods:**

Twelve single nucleotide polymorphisms (SNPs) within novel or unpublished loci with *P*-value < 1.00 × 10^−02^ were selected for genotype with a total of 2,442 cases and 2,798 controls(including 1,156 cases and 2,330 controls from central China, 1,012 cases and 335 controls from southern China and 274 cases and 133 controls from northern China) using Sequenom Massarry system. Associaton analyses were performed using logistic regression with sample region as a covariate through PLINK 1.07 software.

**Results:**

Combined analysis in discovery and central validation dataset discovered a novel locus rs5914778 within *LINC01420* associated with SLE at genome-wide significance (*P* = 1.00 × 10^−08^; odds ratio (OR) = 1.32). We also confirmed rs5914778 in the southern Chinese sample cohort (*P* = 5.31 × 10^−05^; OR = 1.51), and meta-analysis of the samples from the discovery, central and southern validations regions provided robust evidence for the association of rs5914778 (*P* = 5.26 × 10^−12^; OR = 1.35). However, this SNP did not show association with SLE in the northern sample (*P* = 0.33). Further analysis represent the association of northern was significantly heterogeneous compared to central and southern respectively.

**Conclusions:**

Our study increases the number of established susceptibility loci for SLE in Han Chinese population and has further demonstrated the important role of X-linked genetic risk variants in the pathogenesis of SLE in Chinese Han population.

**Electronic supplementary material:**

The online version of this article (doi:10.1186/s13075-015-0857-1) contains supplementary material, which is available to authorized users.

## Introduction

Systemic lupus erythematosus (SLE) is a systemic autoimmune disease that affects predominantly women aged 15–40 years, particularly women of child-bearing age. SLE has been estimated to affect 31–70 cases per 100,000 people in China [1] with a ratio of 9:1 between female and male patients. It is well known that both genetic and environmental factors contribute to disease susceptibility [2–6]. Numerous variants on autosomal loci have been found to be associated with SLE in multiple ethnic groups through candidate gene and genome-wide association studies (GWASs) [7]. Despite the advances in the genetic studies over recent years, the pathogenesis of SLE remains poorly understood.

Because of the huge gender difference in disease prevalence, involvement of the genetic variants on the X chromosome has long been suspected. In recent years, genetic variants of several genes on the X chromosome, such as *MECP2*, *IRAK1*, *TLR7*, and *PRPS2*, have been confirmed to be associated with SLE [8–11]. In particular, single nucleotide polymorphism (SNP) rs3853839 on the 3′ untranslated region (UTR) of *TLR7* was shown to be associated with SLE, especially in Chinese and Japanese male subjects compared with females [10], and a fine mapping study by Kaufman et al. [11] in four different ancestral groups suggested that the nonsynonymous SNP rs1059702 (S196F) within *IRAK1* might be a causal risk variant for SLE. More recently, we performed a meta-analysis of GWASs in Chinese Han populations and followed up the top findings in four additional Asian cohorts [12]. Besides confirming the previously reported associations within *IRAK1-MECP2* (rs1059702) and *L1CAM-MECP2* loci, we also identified a genetic variant (rs7062536) in *PRPS2* on Xp22.3 as a novel susceptibility locus and novel independent associations within the *NAA10* (rs2070028) and *TMEM187* (rs17422) loci.

In this study, with the aim to discover additional X-linked genetic risk variants for SLE, we performed a follow-up study of our previously published GWAS dataset by improving the coverage of genetic variation through imputation and validating the top findings in an additional three independent Chinese Han sample collections [13]. We discovered a novel susceptibility locus *LINC01420* on Xp11.21 associated with SLE.

## Methods

### Subjects

SLE cases and controls were all female and were recruited from multiple hospitals in three geographic regions of China (central, southern, and northern China). All subjects were of self-reported Chinese Han origin. Samples in the GWAS discovery stage (1017 SLE cases and 539 controls) were recruited from central China [13]. Samples in the replication studies were recruited from multiple regions in China, mainly from central (replication: 1156 cases and 2330 controls), southern (replication: 1012 cases and 335 controls), and northern (replication: 274 cases and 133 controls) China. All patients were diagnosed as cases by at least two experienced physicians using the American College of Rheumatology (ACR) criteria revised in 1997 [14]. Controls also were geographically and ethnically matched and clinically evaluated to be without SLE, autoimmune disorders, or family history of autoimmune diseases. Clinical information for all patients and controls was collected through a structured questionnaire. Written informed consent was acquired from all participants. This study was approved by the Institutional Ethical Committee of The First Affiliated Hospital of Anhui Medical University, China–Japan Friendship Hospital, Jiangmen Central Hospital, and The Third Affiliated Hospital of Sun Yat-Sen University, according to Declaration of Helsinki principles. The information for all subjects is summarized in Table 1.

**Table 1:** Summary of samples used in GWASs and replication studies

	Cases				Controls		
Stage	Sample size	Mean (SD) age	Mean (SD) age of onset	Sex (female)	Sample size	Mean (SD) age	Sex (female)
GWAS	1017	34.19 (11.47)	29.84 (10.96)	1017	539	34.31 (12.49)	539
Replication central	1156	35.19 (12.16)	30.70 (11.32)	1156	2330	29.34 (11.20)	2330
Replication southern	1012	33.01 (10.82)	28.22 (10.03)	1012	335	32.59 (13.21)	335
Replication northern	274	35.64 (12.02)	31.10 (11.79)	274	133	29.48 (15.59)	133
Total	3459	34.29 (11.60)	29.76 (10.94)	3459	3337	30.47 (11.98)	3337

GWAS stage samples are from central China

*GWAS* genome-wide association study, *SD* standard deviation

### Genotyping

The genotyping in the discovery stage for the central China cohort was conducted by Illumina 610-Quad Human Beadchip array (Illumina, Inc., San Diego, CA, USA). The genomic DNA was isolated from peripheral blood mononuclear cells (PBMCs) with standard procedures using Flexi Gene DNA kits (QIAGEN GmbH, Hilden, Germany) and was diluted to working concentrations of 50 ng/μl for genome-wide genotyping and 15–20 ng/μl for the validation study. The SNPs in the X chromosome for the validation stage were genotyped using the Sequenom MassArray iPlex Gold platform (Sequenom, Inc., San Diego, CA, USA).

### Statistical analyses

Quality control criteria were applied to genotyped SNPs, and those with minor allele frequency (MAF) <5 % in cases and controls were excluded. SNPs with a genotype missing rate >10 % or Hardy–Weinberg equilibrium (HWE) *P* <3.14 × 10^−6^ in controls were also excluded. Association analysis was performed in PLINK v1.07 [15] using the logistic regression test. We selected 12 SNPs within novel or unpublished loci with *P* <1.00 × 10^−2^ for further validation in 2442 cases and 2798 controls (SNP missing rate <10 % and HWE for female controls with *P* >1.00 × 10^−2^).

To control the impact of population stratification in the validation and combined analysis, we matched cases and controls in terms of ethnic and geographic origins as independent validation samples for combined analysis. Fixed-effects meta-analysis of the four independent studies in the discovery GWAS and three validation cohorts (central, southern and northern) was performed using the inverse variants weighted effect size method in Metasoft version 2.0.0 [16].

We performed the combined analysis of the central region (both discovery and central validation) cohort, southern validation cohort, and northern validation cohort using fixed-effects meta-analysis. The *I*^2^ heterogeneity statistic shows the heterogeneity across studies, with *I*^2^ < 50 and *P*_het_ >0.05 considered insignificant (Table 2).

**Table 2:** Validation of top SNP rs5914778 in southern and northern regions of China

Central meta (discovery + central validation)^a^				Southern validation^b^				Northern validation^c^				Meta (discovery + central + southern validations)^d^				Meta (discovery + central + southern + northern validations)^e^			
Marker	Gene symbol	PVAL (FE)	OR (FE)	PVAL	OR	MAF (case)	MAF (control)	PVAL	OR	MAF (case)	MAF (control)	PVAL (FE)	*I* ^2^ (FE)	PVAL (Het)	OR (FE)	PVAL (FE)	*I* ^2^ (FE)	PVAL (Het)	OR (FE)
rs5914778	*LINC01420*	1.00 × 10^–8^	1.32	5.31 × 10^–5^	1.51	0.352	0.265	3.28 × 10^–1^	0.85	0.252	0.284	5.26 × 10^–12^	0	4.62 × 10^–1^	1.35	1.22 × 10^–10^	65.3	3.44 × 10^–2^	1.31

^a^Consists of 2173 cases and 2869 controls

^b^Consists of 1012 cases and 335 controls

^c^Consists of 274 cases and 133 controls

^d^Consists of 3185 cases and 3204 controls

^e^Consists of 3459 cases and 3337 controls

*FE* fixed effects, *Het* heterogeneity, *I* ^*2*^ heterogeneity statistic, *MAF* minor allele frequency, *OR* odds ratio, *PVAL P* value, *SNP* single nucleotide polymorphism

### Imputation

**Fig. 1:**
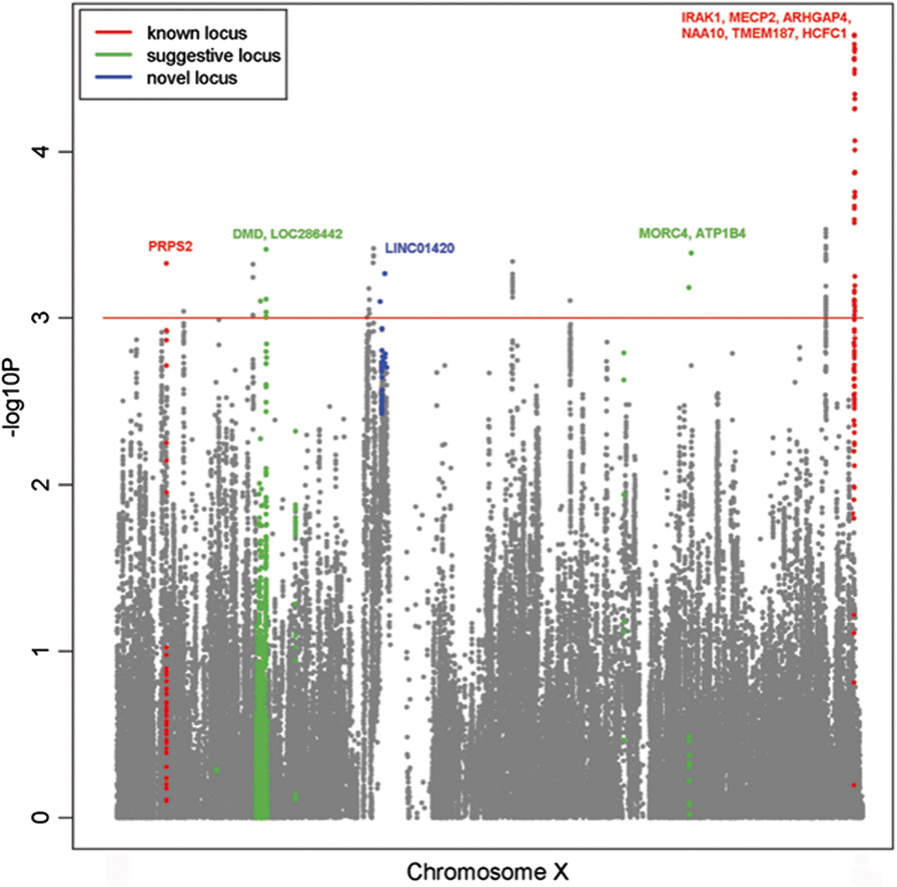
Manhattan plot of the X chromosome association analysis on SLE. Manhattan plot of association results (−log_10_(*P* value)) are depicted with regards to the physical location of SNPs and include both imputed and genotyped association results. Positions and genes were based on National Center for Biotechnology Information build 37 (http://www.ncbi.nlm.nih.gov/assembly/GCF_000001405.25/). The color of gene labeling corresponds to the gene type of known loci, suggestive loci, and novel loci. Known loci are defined as loci published previously, whereby novel loci are defined as loci in the current study validated at genome-wide significance (*P* <5 × 10^−8^)

Imputation of the X chromosome SNPs was performed on the discovery dataset for female individuals using X chromosome nonpseudoautosomal region data from the 1000 Genomes project (phase 1 integrated version 3) as reference [17]. As part of the quality control, SNPs with accuracy score <0.8, missing rate >10 %, MAF <5 % in cases and controls, or HWE *P* <2.89 × 10^−7^ in controls were also excluded. Association was carried out by logistic regression test. The imputation results show that there is no substantial improvement of significant signals between imputed or genotyped SNPs (Fig. 1). No imputed SNPs show better *P* values that would warrant further validation on top of the genotyped SNPs. Therefore, we proceeded with the validation of the selected genotyped SNPs which resided in novel regions.

## Results

### X chromosome discovery and first-stage study

We conducted X chromosome association tests of SLE in the GWAS dataset which consists of 1017 cases and 539 controls, after stringent quality control filtering (see Statistical analyses). The discovery analysis revealed strong evidence of association for all previously identified susceptibility loci on the X chromosome and suggested additional novel risk loci (Additional file 1: Table S1).

To further investigate the observed associations, we imputed the genotypes of additional SNPs that were not genotyped using IMPUTE (v2.0) (Oxf, Oxford, Oxon, UK). After stringent quality control filtering (imputation), no imputed SNPs show better *P* values that would warrant further validation on top of the genotyped SNPs. Therefore, to validate the findings from the discovery analysis, we selected the top SNPs from 14 independent new loci with suggestive association with SLE (*P* <10^−2^) for a follow-up analysis in an additional 1156 cases and 2330 controls of Chinese Han descent from central China. Of the 12 successfully genotyped SNPs, two showed association at *P* <0.05 in the validation samples and six showed consistent effects between the discovery and validation samples. The meta-analysis results for the 12 SNPs in the combined discovery and central validation dataset totaling 2173 cases and 2869 controls, using fixed-effects and random-effects models, are presented in Table 3. The combined analysis discovered a novel locus rs5914778 within *LINC01420* associated with SLE disease at genome-wide significance (*P* = 1.00 × 10^−8^; odds ratio (OR) = 1.32).

**Table 3:** Validation of selected SNPs on the X chromosome in the central region

					Discovery set (central)^a^				Central validation^b^					Meta (discovery + central validation)^c^				
Marker	Minor allele	Position (Hg19)	Annotation	Gene symbol	PVAL	OR	MAF case	MAF control	PVAL	OR	MAF case	MAF control	PVAL (FE)	PVAL (RE)	OR (FE)	OR (RE)	*I* ^2^	PVAL (Het)
rs5914778	A	56758231	Intronic	*LINC01420*	3.59 × 10^–3^	1.29	0.286	0.238	7.15 × 10^–7^	1.33	0.296	0.24	1.00 × 10^–8^	1.00 × 10^–8^	1.32	1.32	0	7.58 × 10^–1^
rs1527803	A	16421982	184 kb 3′ of CTPS2	NA	6.75 × 10^–2^	0.87	0.368	0.402	1.90 × 10^–2^	1.13	0.403	0.374	3.63 × 10^–1^	9.82 × 10^–1^	1.04	1.00	87.5	4.70 × 10^–3^
rs5963635	A	39166975	None	*LOC286442*	1.40 × 10^–3^	1.34	0.247	0.197	2.17 × 10^–1^	1.08	0.230	0.217	5.17 × 10^–3^	1.08 × 10^–1^	1.15	1.19	74.1	4.94 × 10^–2^
rs1860995	G	119502122	Intronic	*ATP1B4*	7.48 × 10^–4^	1.30	0.417	0.355	6.74 × 10^–1^	1.02	0.395	0.389	1.69 × 10^–2^	2.87 × 10^–1^	1.10	1.11	81.4	4.33 × 10^–2^
rs2536576	G	147417249	165 kb 5′ of AFF2	NA	5.73 × 10^–3^	1.34	0.172	0.134	9.43 × 10^–1^	1.01	0.152	0.152	1.12 × 10^–1^	3.36 × 10^–1^	1.10	1.15	80.2	2.45 × 10^–2^
rs17326228	G	106239318	Intronic	*MORC4*	2.56 × 10^–4^	1.33	0.438	0.370	3.01 × 10^–1^	0.95	0.418	0.432	3.82 × 10^–1^	6.14 × 10^–1^	1.02	1.05	88.5	1.75 × 10^–3^
rs17329976	G	142130195	8.1 KB 3′ of SPANXN4	NA	4.28 × 10^–2^	0.83	0.179	0.209	1.58 × 10^–1^	0.91	0.160	0.174	2.23 × 10^–2^	3.62 × 10^–2^	0.85	0.83	51.6	1.62 × 10^–1^
rs5936901	G	67975499	30 kb 3′ of STARD8	NA	4.45 × 10^–4^	1.33	0.371	0.308	5.53 × 10^–1^	0.97	0.342	0.350	1.28 × 10^–1^	4.50 × 10^–1^	1.07	1.13	90.3	1.30 × 10^–3^
rs10218247	A	83476018	33 kb 5′ of RPS6KA6	NA	3.67 × 10^–3^	0.78	0.241	0.289	5.47 × 10^–1^	0.96	0.248	0.255	2.85 × 10^–2^	3.06 × 10^–1^	0.91	0.86	82.4	1.24 × 10^–2^
rs5970959	G	22987793	30 kb 5′ of DDX53	*DDX53*	1.42 × 10^–2^	1.25	0.254	0.215	1.57 × 10^–1^	0.92	0.251	0.267	3.76 × 10^–1^	6.41 × 10^–1^	1.03	1.05	86.7	2.06 × 10^–3^
rs6631753	A	33199451	Intronic	*DMD*	6.94 × 10^–3^	0.81	0.376	0.426	5.09 × 10^–1^	1.04	0.374	0.366	7.12 × 10^–2^	4.69 × 10^–1^	0.93	0.91	89.2	4.35 × 10^–3^
rs2411864	G	16377763	206 kb 3′ of GRPR	NA	5.04 × 10^–3^	0.76	0.154	0.194	4.81 × 10^–1^	1.05	0.165	0.159	2.98 × 10^–1^	5.16 × 10^–1^	0.94	0.90	86.2	7.10 × 10^–3^

^a^From Han et al. [13] with 1017 cases and 539 controls from central China

^b^Consists of 1156 cases and 2330 controls

^c^Consists of 2173 cases and 2869 controls

*FE* fixed effects, *Het* heterogeneity, *I* ^*2*^ heterogeneity statistic, *MAF* minor allele frequency, *NA* not available, *OR* odds ratio, *PVAL P* value, *RE* random effects, *SNP* single nucleotide polymorphism

### Further replication of selected SNPs and the heterogeneity test

We performed further replication analysis of rs5914778 in two additional independent samples of Chinese Han descent from the southern and northern regions of China. The replication in the southern Chinese sample cohort, consisting of a total of 1012 cases and 335 controls, provided strong supporting evidence for the association of rs5914778 with SLE (*P* = 5.31 × 10^−5^; OR = 1.51). The meta-analysis of the samples from the central and southern regions, totaling 3185 cases and 3204 controls, provided robust evidence for the association of rs5914778 (*P* = 5.26 × 10^−12^; OR = 1.35). In addition, the strength of the association is very consistent without any evidence of heterogeneity (*P*_het_ = 0.46, *I*^2^ = 0) (Table 2).

Intriguingly, this SNP did not show association with SLE in the northern sample with a total of 274 cases and 133 controls (*P* = 0.33, OR = 0.85), and the SNP actually showed an opposite effect in the northern sample as compared with the central and southern samples (Table 2). This could be because of the very small sample size of the northern replication cohort. Further studies are needed to confirm the heterogeneity of this association between the northern and central/southern Chinese populations.

**Fig. 2:**
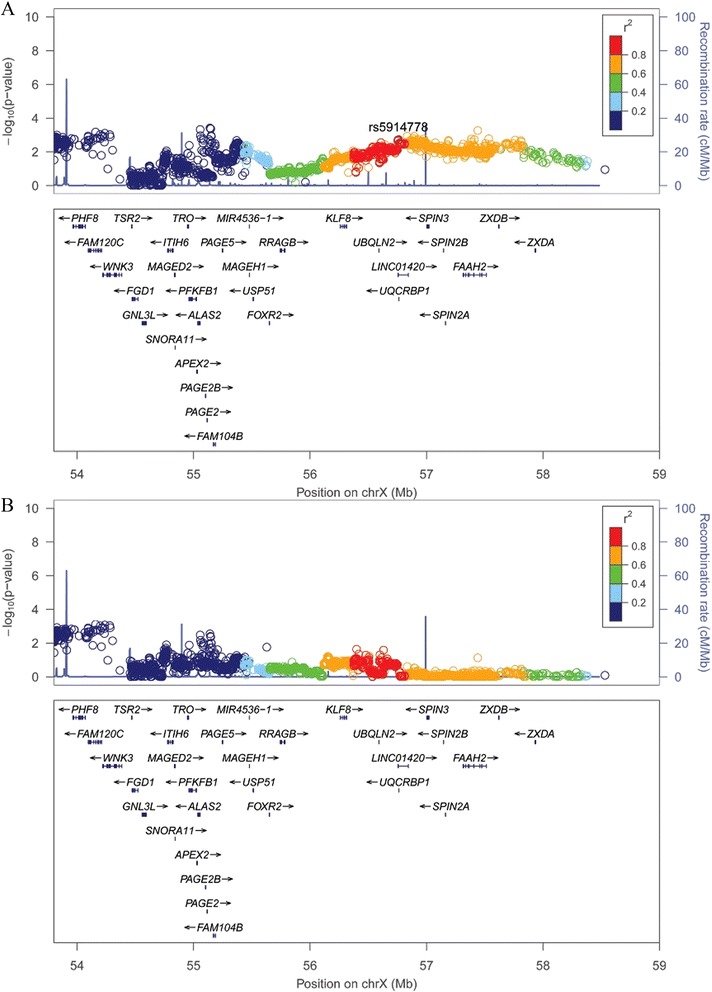
**a** Regional association plots of new loci (Xp11.21, *LINC01420*). The association results (−log_10_(*P* value)) of SNPs from the discovery analysis were shown against their map positions (NCBI build 37). Validated SNP rs5914778 is labeled *purple*. **b** Regional association plots after conditioning on rs5914778. The association results (−log_10_(*P* value)) of SNPs from the discovery analysis after conditioning on rs5914778. All map coordinates are based on NCBI build 37. *chrX* X chromosome, *NCBI* National Center for Biotechnology Information

Lastly, we performed a joint analysis for all of the discovery, central, southern, and northern validation samples totaling 3459 cases and 3337 controls, using a fixed-effects meta-analysis. The association at rs5914778 (*LINC01420*) on Xp11.21 surpassed the genome-wide significance (*P* = 1.22 × 10^−10^; OR = 1.31), but a moderate heterogeneity of association was observed within the samples (*P*_het_ = 0.034, *I*^2^ = 65.3) (Table 2 and Fig. 2).

## Discussion

Through the discovery and validation analyses in two independent female samples from the central region of China, we have discovered a novel SLE susceptibility locus at rs5914778 (*LINC01420*) on Xp11.21 at the genome-wide significance. Further replication analysis in the independent sample of southern Chinese confirmed the association with strong evidence. The analysis of the independent sample of northern Chinese failed to replicate the association, but the sample size of the northern cohort is very small.

rs5914778 is located within a long intronic region between the first and second exons of *LINC01420. LINC01420* is a long noncoding RNA with enhancers marked by histone modifications in human umbilical vein endothelial cells (HUVEC) and HSMM based on HaploReg annotation [18] (Additional file 2: Table S2). *LINC01420* was found to have sex-specific DNAse I hypersensitivity patterns which showed H3K4me3 histone enrichment and strong expression in females only [19]. According to the regulatory annotation information from the ENCODE project [20], this SNP is within a DNase I hypersensitive site that was detected in the lymphoblastoid cell line. *LINC01420* may maintain the X inactivation which avoids X-linked gene overexpression through dosage compensation in females [21]. Long noncoding RNAs have been shown to be associated with many complex diseases such as psoriasis, breast cancer, gastric cancer, colorectal cancer, osteosarcoma, adrenocortical cancer, and cardiovascular diseases in recent years [22–28]. Some noncoding RNAs also play a role in the pathogenesis and progression of hepatocellular carcinoma, and may act as therapeutic targets for hepatocellular carcinoma [29]. In order to reveal whether there are expression difference of *LINC01420* between females and males, we performed gene expression analyses using the gene expression data from CD4^+^ T cells and monocytes from 461 healthy individuals [30] and the gene expression data from PBMCs of 82 controls [31] in GEO datasets. However, we did not obtain the gene expression result of *LINC01420*, indicating that *LINC01420* might express too low to be detected in blood cells from healthy individuals. Hence, more work will be needed to elucidate the biological mechanism through which *LINC01420* influences SLE pathogenesis.

**Fig. 3:**
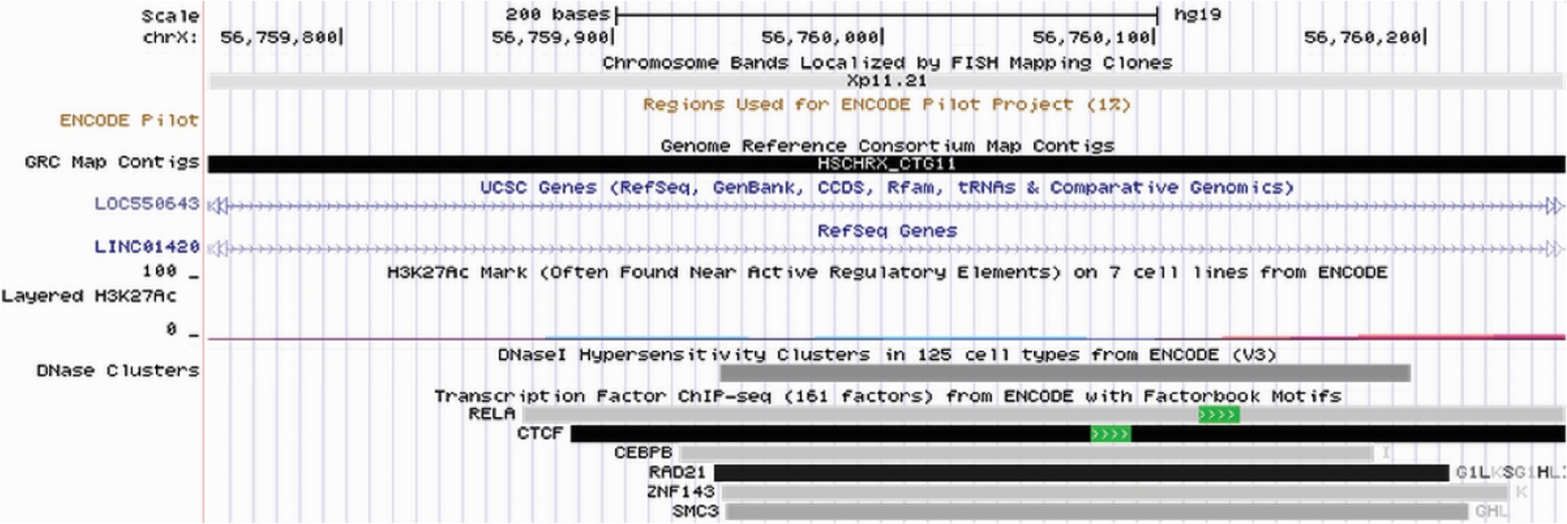
Transcription factor binding sites of rs5913992 SNP (ENCODE). The transcription track from the UCSC (University of California, Santa Cruz) genome browser showed regions where transcription factors responsible for modulating gene transcription bind to DNA as assayed by ChIP-seq

We also observed another SNP, rs5913992, in perfect linkage disequilibrium with our top SNP rs5914778 (*R*^2^ = 1) that was predicted to be functional by Regulome DB (LSJU, Stanford, CA, USA) with a score of 2b (likely to affect binding of motifs, transcription factors, and enhancer histone marks) in this locus [32]. rs5913992 is also within the region of the binding sites of six overlapping transcription factors (transcription factor binding sites)—*RELA*, *CTCF*, *CEBPB*, *RAD21*, *ZNF143*, and *SMC3*—that were detected by ChIP-Seq analysis in lymphoblastoid, epithelial, endothelial, breast cancer, and myeloid leukemia cell lines (Fig. 3). The prediction by Regulome DB indicates that this SNP overlaps a potential consensus *EWSR1-FLI1* binding motif within the binding sites of the six transcription factor binding sites (Additional file 3: Table S3).

We observed the same risk effect at rs5914778 in the central and southern validation results, while the opposite effect was observed in the northern validation results. The association of the northern cohort was significantly heterogeneous compared with the central and southern cohorts respectively (*P*_het_ = 0.034, *I*^2^ = 65.3). Several previous studies have demonstrated differences in disease risk between northern and southern Chinese, and further studies in more northern Chinese samples will be needed to confirm the genetic heterogeneity of this susceptibility locus among the central, southern, and northern Chinese populations [33–35].

## Conclusions

We performed a three-stage X chromosome association analysis of SLE in the Chinese Han population and discovered a novel susceptibility locus on Xp11.21. Although further studies will be required to understand how the locus influences the etiology of SLE, the discovery of this novel locus has further expanded the role of the X chromosome in the development of SLE in the Chinese Han population.

## Additional files

Additional file 1: Table S1. Presenting association results for the X chromosome SLE GWAS cohort. (DOC 241 kb) Additional file 2: Table S2. Presenting HaploReg annotation for rs5914778 (Query SNP: rs5914778 and variants with *r* ^2^ ≧0.8). (DOC 46 kb) Additional file 3: Table S3. Presenting the motifs predicted to be affected by rs5913992 SNP (Regulome DB). (DOC 171 kb)

## Abbreviations

ACR: American College of Rheumatology
GWAS: Genome-wide association study
HUVEC: Human umbilical vein endothelial cells
HWE: Hardy–Weinberg equilibrium
MAF: Minor allele frequency
OR: Odds ratio
PBMC: Peripheral blood mononuclear cell
SLE: Systemic lupus erythematosus
SNP: Single nucleotide polymorphism
UTR: Untranslated region
