# Supplementary material for: Discovery of a novel genetic susceptibility locus on X chromosome for systemic lupus erythematosus
Source: Arthritis Res Ther. 2015 Dec 3;17:349. doi: 10.1186/s13075-015-0857-1 (PMC4669597; doi:10.1186/s13075-015-0857-1)
Supplement: Additional file 1: Table S1. — Presenting association results for the X chromosome SLE GWAS cohort. (DOC 241 kb) [file 13075_2015_857_MOESM1_ESM.doc]

**Additional file 1: Table S1. Association results for chr-X SLE GWAS cohort (Anhui)**

| **SNP** | **Gene** | **Minor Allele** | **OR** | **SE** | **L95** | **U95** | **P-value** |
| --- | --- | --- | --- | --- | --- | --- | --- |
| rs1059702 | IRAK1 | G | 0.72 | 0.09 | 0.61 | 0.85 | 1.34E-04 |
| rs2239464 | MECP2 | G | 0.72 | 0.09 | 0.6 | 0.85 | 1.74E-04 |
| rs2734647 | MECP2 | G | 0.73 | 0.09 | 0.62 | 0.86 | 2.18E-04 |
| rs6631753 | DMD | A | 0.78 | 0.07 | 0.69 | 0.9 | 3.85E-04 |
| rs5956251 | - | A | 0.76 | 0.08 | 0.65 | 0.89 | 4.06E-04 |
| rs5972178 | - | C | 0.8 | 0.07 | 0.7 | 0.91 | 5.68E-04 |
| rs10218247 | - | A | 0.77 | 0.08 | 0.66 | 0.89 | 5.71E-04 |
| rs2536576 | - | G | 1.36 | 0.09 | 1.14 | 1.61 | 6.41E-04 |
| rs1860995 | ATP1B4 | G | 1.26 | 0.07 | 1.1 | 1.44 | 6.53E-04 |
| rs1860814 | - | G | 1.35 | 0.09 | 1.13 | 1.61 | 7.45E-04 |
| rs5914638 | - | G | 1.28 | 0.07 | 1.11 | 1.49 | 7.95E-04 |
| rs2516036 | FAM120C | G | 0.68 | 0.12 | 0.54 | 0.85 | 8.90E-04 |
| rs2266888 | TMEM187 | G | 0.77 | 0.08 | 0.66 | 0.9 | 9.57E-04 |
| rs2495794 | FAM120C | A | 0.68 | 0.12 | 0.54 | 0.86 | 1.09E-03 |
| rs2806010 | MIR548AE1 | G | 1.24 | 0.07 | 1.09 | 1.4 | 1.13E-03 |
| rs17422 | HCFC1 | A | 0.77 | 0.08 | 0.66 | 0.9 | 1.17E-03 |
| rs3761622 | TLR8-AS1 | C | 0.74 | 0.09 | 0.61 | 0.89 | 1.21E-03 |
| rs942273 | MIR548AE1 | C | 1.23 | 0.07 | 1.09 | 1.4 | 1.23E-03 |
| rs1408095 | MIR548AE1 | A | 1.23 | 0.07 | 1.08 | 1.4 | 1.37E-03 |
| rs2495782 | FAM120C | A | 0.69 | 0.12 | 0.55 | 0.87 | 1.59E-03 |
| rs17326228 | MORC4 | G | 1.23 | 0.07 | 1.08 | 1.41 | 1.61E-03 |
| rs5960060 | - | A | 1.23 | 0.07 | 1.08 | 1.4 | 1.66E-03 |
| rs5960395 | PHF8 | A | 0.68 | 0.12 | 0.54 | 0.87 | 1.69E-03 |
| rs17329976 | - | G | 0.77 | 0.09 | 0.65 | 0.91 | 1.75E-03 |
| rs12556165 | - | C | 0.82 | 0.07 | 0.72 | 0.93 | 1.81E-03 |
| rs12688561 | FAM120C | A | 0.69 | 0.12 | 0.55 | 0.87 | 1.82E-03 |
| rs5909765 | - | A | 0.74 | 0.1 | 0.61 | 0.89 | 1.92E-03 |
| rs7062536 | PRPS2 | A | 0.8 | 0.07 | 0.7 | 0.92 | 1.92E-03 |
| rs4288493 | - | G | 1.24 | 0.07 | 1.08 | 1.43 | 1.93E-03 |
| rs6612662 | - | G | 1.24 | 0.07 | 1.08 | 1.43 | 1.96E-03 |
| rs4907832 | - | A | 0.76 | 0.09 | 0.63 | 0.9 | 1.98E-03 |
| rs4535870 | - | C | 1.24 | 0.07 | 1.08 | 1.43 | 1.98E-03 |
| rs5914778 | LINC01420 | A | 1.25 | 0.07 | 1.09 | 1.44 | 2.00E-03 |
| rs5914860 | - | C | 1.24 | 0.07 | 1.08 | 1.43 | 2.09E-03 |
| rs5936901 | - | G | 1.23 | 0.07 | 1.08 | 1.41 | 2.11E-03 |
| rs17267184 | RPS6KA6 | A | 0.74 | 0.1 | 0.62 | 0.9 | 2.11E-03 |
| rs1343096 | - | A | 0.74 | 0.1 | 0.62 | 0.9 | 2.15E-03 |
| rs1560514 | FAAH2 | A | 1.24 | 0.07 | 1.08 | 1.42 | 2.16E-03 |
| rs1323751 | MIR548AE1 | A | 1.22 | 0.07 | 1.07 | 1.38 | 2.18E-03 |
| rs17281143 | - | G | 1.26 | 0.08 | 1.09 | 1.47 | 2.22E-03 |
| rs6418619 | - | A | 0.8179 | 0.06579 | 0.7189 | 0.9305 | 2.25E-03 |
| rs6612720 | - | A | 1.24 | 0.07048 | 1.08 | 1.424 | 2.25E-03 |
| rs2026622 | LINC01420 | A | 1.24 | 0.07049 | 1.08 | 1.424 | 2.27E-03 |
| rs5914806 | - | G | 1.24 | 0.07049 | 1.08 | 1.424 | 2.27E-03 |
| rs6616617 | MORC4 | A | 1.225 | 0.06666 | 1.075 | 1.396 | 2.34E-03 |
| rs2532869 | - | C | 0.819 | 0.0658 | 0.7199 | 0.9318 | 2.41E-03 |
| rs5960810 | - | A | 1.239 | 0.07051 | 1.079 | 1.422 | 2.41E-03 |
| rs5970959 | PTCHD1-AS | G | 1.26 | 0.08 | 1.09 | 1.47 | 2.42E-03 |
| rs6638625 | - | A | 1.244 | 0.07201 | 1.08 | 1.433 | 2.43E-03 |
| rs6521788 | - | G | 0.6944 | 0.1205 | 0.5483 | 0.8793 | 2.46E-03 |
| rs5960612 | PHF8 | A | 0.6924 | 0.1216 | 0.5456 | 0.8788 | 2.51E-03 |
| rs5961058 | - | A | 0.8215 | 0.06511 | 0.7231 | 0.9333 | 2.53E-03 |
| rs4379572 | - | G | 1.236 | 0.07041 | 1.077 | 1.419 | 2.60E-03 |
| rs5914776 | - | A | 1.244 | 0.07272 | 1.079 | 1.435 | 2.66E-03 |
| rs5922916 | RPS6KA6 | A | 0.7489 | 0.09626 | 0.6201 | 0.9044 | 2.66E-03 |
| rs2411864 | - | G | 0.77 | 0.09 | 0.64 | 0.91 | 2.67E-03 |
| rs3764880 | TLR8 | A | 0.7549 | 0.09364 | 0.6283 | 0.9069 | 2.67E-03 |
| rs6611574 | - | A | 1.235 | 0.07046 | 1.076 | 1.418 | 2.75E-03 |
| rs1527803 | - | A | 0.82 | 0.07 | 0.72 | 0.93 | 2.84E-03 |
| rs6529663 | - | G | 1.24 | 0.07 | 1.08 | 1.43 | 3.03E-03 |
| rs4826508 | LINC01420 | G | 1.233 | 0.0708 | 1.073 | 1.417 | 3.06E-03 |
| rs2335517 | - | A | 1.301 | 0.08897 | 1.093 | 1.549 | 3.09E-03 |
| rs5914037 | - | A | 1.231 | 0.07035 | 1.073 | 1.413 | 3.12E-03 |
| rs4843993 | - | G | 1.295 | 0.08755 | 1.091 | 1.538 | 3.14E-03 |
| rs5915082 | - | A | 1.23 | 0.07 | 1.07 | 1.42 | 3.21E-03 |
| rs5936343 | - | A | 1.294 | 0.08755 | 1.09 | 1.536 | 3.24E-03 |
| rs5944365 | - | A | 1.286 | 0.0856 | 1.087 | 1.521 | 3.32E-03 |
| rs3788935 | TLR8 | A | 0.7606 | 0.09322 | 0.6336 | 0.913 | 3.33E-03 |
| rs11094927 | - | A | 1.214 | 0.066 | 1.066 | 1.381 | 3.34E-03 |
| rs995154 | - | A | 1.21 | 0.07 | 1.07 | 1.38 | 3.39E-03 |
| rs5960307 | - | G | 0.8266 | 0.06505 | 0.7277 | 0.939 | 3.43E-03 |
| rs5914785 | LINC01420 | A | 1.229 | 0.07064 | 1.071 | 1.412 | 3.45E-03 |
| rs5960235 | SPIN3 | G | 1.228 | 0.07034 | 1.07 | 1.41 | 3.48E-03 |
| rs12835268 | - | A | 0.8238 | 0.06637 | 0.7233 | 0.9382 | 3.50E-03 |
| rs5914795 | LINC01420 | A | 1.229 | 0.07079 | 1.07 | 1.412 | 3.59E-03 |
| rs5913993 | LINC01420 | A | 1.227 | 0.07063 | 1.069 | 1.41 | 3.73E-03 |
| rs726441 | - | A | 0.8299 | 0.06438 | 0.7315 | 0.9415 | 3.78E-03 |
| rs5933907 | - | A | 1.268 | 0.08218 | 1.08 | 1.49 | 3.81E-03 |
| rs6641214 | - | A | 1.282 | 0.08586 | 1.083 | 1.517 | 3.81E-03 |
| rs11094877 | - | A | 0.8262 | 0.06599 | 0.726 | 0.9403 | 3.82E-03 |
| rs11091412 | - | A | 1.22 | 0.06888 | 1.066 | 1.396 | 3.88E-03 |
| rs6612746 | SPIN3 | A | 1.225 | 0.07033 | 1.067 | 1.406 | 3.90E-03 |
| rs5978593 | TLR8_AS1 | G | 0.7525 | 0.0986 | 0.6203 | 0.913 | 3.94E-03 |
| rs2269368 | ARHGAP4 | G | 0.817 | 0.07042 | 0.7117 | 0.938 | 4.11E-03 |
| rs3810757 | - | A | 1.269 | 0.08315 | 1.078 | 1.494 | 4.12E-03 |
| rs10854983 | - | G | 0.83 | 0.07 | 0.73 | 0.94 | 4.16E-03 |
| rs6612721 | - | A | 1.225 | 0.07073 | 1.066 | 1.407 | 4.16E-03 |
| rs5925798 | - | A | 0.82 | 0.07 | 0.71 | 0.94 | 4.17E-03 |
| rs7884579 | - | G | 1.213 | 0.06736 | 1.063 | 1.384 | 4.18E-03 |
| rs6571303 | TMEM187 | G | 0.8068 | 0.07537 | 0.696 | 0.9352 | 4.39E-03 |
| rs2056918 | - | G | 0.7275 | 0.1118 | 0.5844 | 0.9058 | 4.44E-03 |
| rs5966868 | - | A | 0.7437 | 0.1041 | 0.6064 | 0.912 | 4.44E-03 |
| rs7883778 | - | G | 0.6869 | 0.132 | 0.5303 | 0.8897 | 4.44E-03 |
| rs5925786 | - | A | 0.8207 | 0.0696 | 0.7161 | 0.9407 | 4.53E-03 |
| rs9306569 | - | G | 0.83 | 0.06572 | 0.7297 | 0.9441 | 4.58E-03 |
| rs7065919 | DMD | G | 0.8288 | 0.06625 | 0.7279 | 0.9438 | 4.61E-03 |
| rs5914036 | SPIN3 | A | 1.22 | 0.07032 | 1.063 | 1.4 | 4.69E-03 |
| rs5960936 | - | A | 0.8294 | 0.06617 | 0.7285 | 0.9442 | 4.70E-03 |
| rs5963635 | LOC286442 | A | 1.25 | 0.08 | 1.07 | 1.45 | 4.76E-03 |
| rs6617836 | - | A | 0.7956 | 0.081 | 0.6788 | 0.9325 | 4.76E-03 |
| rs1342219 | - | A | 0.7278 | 0.1128 | 0.5835 | 0.9079 | 4.85E-03 |
| rs6622208 | - | G | 1.206 | 0.0665 | 1.059 | 1.374 | 4.85E-03 |
| rs5918209 | CASK | G | 0.7832 | 0.08709 | 0.6603 | 0.9289 | 5.01E-03 |
| rs6523960 | - | G | 1.208 | 0.06739 | 1.058 | 1.378 | 5.08E-03 |
| rs5924847 | - | C | 1.251 | 0.08007 | 1.07 | 1.464 | 5.10E-03 |
| rs6617830 | - | A | 0.7973 | 0.08095 | 0.6803 | 0.9344 | 5.13E-03 |
| rs1323757 | - | A | 0.8303 | 0.0665 | 0.7288 | 0.9458 | 5.15E-03 |
| rs5914893 | - | G | 1.22 | 0.07 | 1.06 | 1.4 | 5.17E-03 |
| rs3859913 | - | G | 1.204 | 0.06631 | 1.057 | 1.371 | 5.20E-03 |
| rs2890089 | - | C | 1.218 | 0.07064 | 1.061 | 1.399 | 5.21E-03 |
| rs5936206 | - | C | 1.279 | 0.08811 | 1.076 | 1.52 | 5.25E-03 |
| rs5913850 | - | A | 0.8319 | 0.06613 | 0.7308 | 0.947 | 5.38E-03 |
| rs1937249 | - | A | 1.207 | 0.06752 | 1.057 | 1.377 | 5.39E-03 |
| rs5916449 | - | G | 1.215 | 0.07017 | 1.059 | 1.395 | 5.43E-03 |
| rs12013552 | - | C | 1.215 | 0.07019 | 1.059 | 1.395 | 5.46E-03 |
| rs5921138 | - | A | 0.7492 | 0.1042 | 0.6109 | 0.9189 | 5.57E-03 |
| rs2982249 | - | A | 1.217 | 0.07074 | 1.059 | 1.398 | 5.58E-03 |
| rs11795541 | - | G | 1.237 | 0.07672 | 1.064 | 1.438 | 5.59E-03 |
| rs5925802 | - | A | 0.8248 | 0.06955 | 0.7197 | 0.9452 | 5.60E-03 |
| rs5977894 | - | A | 1.207 | 0.06809 | 1.057 | 1.38 | 5.63E-03 |
| rs2188615 | - | A | 1.196 | 0.06468 | 1.053 | 1.358 | 5.69E-03 |
| rs2188616 | - | A | 1.196 | 0.06468 | 1.053 | 1.358 | 5.69E-03 |
| rs1925926 | GDPD2 | C | 0.8097 | 0.07638 | 0.6971 | 0.9405 | 5.72E-03 |
| rs7055735 | DACH2 | A | 0.8101 | 0.07623 | 0.6977 | 0.9407 | 5.75E-03 |
| rs5936524 | EDA | C | 1.216 | 0.07088 | 1.058 | 1.397 | 5.78E-03 |
| rs2214563 | - | G | 1.234 | 0.07617 | 1.063 | 1.432 | 5.83E-03 |
| rs7877755 | - | A | 0.7225 | 0.1179 | 0.5734 | 0.9103 | 5.83E-03 |
| rs5975417 | - | A | 1.206 | 0.06801 | 1.055 | 1.378 | 5.89E-03 |
| rs4403537 | - | A | 0.7515 | 0.1039 | 0.6131 | 0.9212 | 5.96E-03 |
| rs9699111 | - | A | 0.8354 | 0.06549 | 0.7348 | 0.9498 | 6.02E-03 |
| rs17344059 | - | G | 0.8153 | 0.07438 | 0.7047 | 0.9432 | 6.04E-03 |
| rs5926470 | - | A | 1.202 | 0.06693 | 1.054 | 1.37 | 6.04E-03 |
| rs6654792 | - | G | 1.259 | 0.08376 | 1.068 | 1.483 | 6.04E-03 |
| rs2285563 | ARX | C | 1.195 | 0.06493 | 1.052 | 1.357 | 6.06E-03 |
| rs5914994 | FAAH2 | A | 1.212 | 0.06996 | 1.056 | 1.39 | 6.09E-03 |
| rs5914700 | - | G | 1.217 | 0.07176 | 1.058 | 1.401 | 6.10E-03 |
| rs5986613 | - | G | 1.262 | 0.08477 | 1.069 | 1.49 | 6.10E-03 |
| rs697664 | - | G | 1.212 | 0.07009 | 1.056 | 1.39 | 6.15E-03 |
| rs1467342 | - | G | 1.212 | 0.07022 | 1.056 | 1.391 | 6.17E-03 |
| rs5959353 | - | G | 0.7367 | 0.1117 | 0.5919 | 0.9169 | 6.21E-03 |
| rs5953534 | - | A | 1.327 | 0.1035 | 1.083 | 1.625 | 6.28E-03 |
| rs765076 | - | G | 0.8179 | 0.07362 | 0.708 | 0.9449 | 6.33E-03 |
| rs707346 | SPIN2B | A | 1.211 | 0.07017 | 1.055 | 1.39 | 6.35E-03 |
| rs5928345 | IL1RAPL1 | C | 1.272 | 0.08823 | 1.07 | 1.512 | 6.48E-03 |
| rs5961051 | - | A | 0.8357 | 0.06593 | 0.7344 | 0.951 | 6.48E-03 |
| rs2808725 | - | A | 1.192 | 0.06468 | 1.05 | 1.353 | 6.53E-03 |
| rs5914902 | - | A | 1.21 | 0.07 | 1.05 | 1.39 | 6.61E-03 |
| rs5978005 | - | G | 1.201 | 0.06746 | 1.052 | 1.371 | 6.69E-03 |
| rs4826580 | - | A | 1.211 | 0.07053 | 1.054 | 1.39 | 6.73E-03 |
| rs5908660 | - | G | 1.317 | 0.1024 | 1.078 | 1.61 | 7.14E-03 |
| rs5977810 | - | G | 1.201 | 0.06814 | 1.051 | 1.373 | 7.14E-03 |
| rs859603 | SASH3 | G | 1.259 | 0.08565 | 1.064 | 1.489 | 7.20E-03 |
| rs5960434 | - | A | 1.196 | 0.06686 | 1.049 | 1.364 | 7.32E-03 |
| rs209764 | NDP | A | 0.8377 | 0.0661 | 0.7359 | 0.9535 | 7.36E-03 |
| rs7059234 | - | G | 1.19 | 0.06489 | 1.048 | 1.351 | 7.37E-03 |
| rs6610903 | EFHC2 | A | 1.322 | 0.1045 | 1.077 | 1.623 | 7.51E-03 |
| rs1266322 | - | A | 1.21 | 0.0714 | 1.052 | 1.392 | 7.57E-03 |
| rs5986629 | - | G | 1.243 | 0.08142 | 1.059 | 1.458 | 7.61E-03 |
| rs5911059 | - | G | 0.8408 | 0.06498 | 0.7403 | 0.955 | 7.62E-03 |
| rs512119 | - | G | 1.203 | 0.06939 | 1.05 | 1.379 | 7.67E-03 |
| rs6628425 | IL1RAPL1 | G | 1.226 | 0.07657 | 1.055 | 1.424 | 7.83E-03 |
| rs6627929 | - | A | 0.8312 | 0.06956 | 0.7253 | 0.9526 | 7.85E-03 |
| rs2280964 | CXCR3 | A | 1.198 | 0.06807 | 1.048 | 1.369 | 7.93E-03 |
| rs6527265 | DMD | A | 0.838 | 0.0666 | 0.7355 | 0.9549 | 7.98E-03 |
| rs5923562 | DACH2 | A | 1.204 | 0.06999 | 1.05 | 1.381 | 8.03E-03 |
| rs5914734 | - | A | 1.209 | 0.07152 | 1.05 | 1.39 | 8.08E-03 |
| rs723556 | ARAF | G | 0.84 | 0.06586 | 0.7383 | 0.9557 | 8.10E-03 |
| rs6617168 | - | G | 1.192 | 0.06661 | 1.046 | 1.358 | 8.39E-03 |
| rs5928201 | DMD | A | 0.7415 | 0.1136 | 0.5935 | 0.9265 | 8.49E-03 |
| rs2071251 | ZNF185 | A | 0.8348 | 0.06871 | 0.7296 | 0.9552 | 8.60E-03 |
| rs6521411 | - | C | 1.206 | 0.0713 | 1.049 | 1.387 | 8.60E-03 |
| rs7876155 | FRMPD4 | A | 0.7902 | 0.08973 | 0.6628 | 0.9422 | 8.69E-03 |
| rs5955456 | - | G | 1.237 | 0.08124 | 1.055 | 1.451 | 8.75E-03 |
| rs5942373 | - | C | 0.8139 | 0.07877 | 0.6975 | 0.9498 | 8.96E-03 |
| rs12009868 | - | A | 1.193 | 0.06744 | 1.045 | 1.361 | 8.97E-03 |
| rs1489965 | - | G | 1.205 | 0.07143 | 1.048 | 1.386 | 9.05E-03 |
| rs5975460 | - | A | 1.192 | 0.06753 | 1.045 | 1.361 | 9.14E-03 |
| rs4830593 | KAL1 | A | 1.207 | 0.0723 | 1.048 | 1.391 | 9.16E-03 |
| rs5924783 | - | G | 0.8361 | 0.06867 | 0.7308 | 0.9566 | 9.17E-03 |
| rs5924779 | ZNF185 | G | 0.8361 | 0.06871 | 0.7308 | 0.9567 | 9.20E-03 |
| rs5911011 | - | A | 0.7329 | 0.1196 | 0.5798 | 0.9266 | 9.38E-03 |
| rs1860012 | - | G | 0.8413 | 0.0666 | 0.7383 | 0.9586 | 9.46E-03 |
| rs5975439 | - | A | 1.192 | 0.06755 | 1.044 | 1.36 | 9.49E-03 |
| rs5930628 | - | A | 1.186 | 0.06585 | 1.043 | 1.35 | 9.54E-03 |
| rs2813808 | - | G | 1.186 | 0.06598 | 1.042 | 1.35 | 9.66E-03 |
| rs5935409 | - | C | 1.239 | 0.08297 | 1.053 | 1.458 | 9.73E-03 |
| rs5923542 | DACH2 | A | 1.2 | 0.07063 | 1.045 | 1.378 | 9.76E-03 |
| rs5962817 | - | A | 1.189 | 0.06727 | 1.042 | 1.357 | 9.98E-03 |
| rs5933555 | KDM5C | A | 0.8124 | 0.08067 | 0.6936 | 0.9515 | 9.99E-03 |
