# Supplementary material for: Discovery of a novel genetic susceptibility locus on X chromosome for systemic lupus erythematosus
Source: Arthritis Res Ther. 2015 Dec 3;17:349. doi: 10.1186/s13075-015-0857-1 (PMC4669597; doi:10.1186/s13075-015-0857-1)
Supplement: Additional file 2: Table S2. — Presenting HaploReg annotation for rs5914778 (Query SNP: rs5914778 and variants with r 2 ≧0.8). (DOC 46 kb) [file 13075_2015_857_MOESM2_ESM.doc]

**Additional file 2: Table S2. HaploReg annotation for** [**rs5914778**](http://www.broadinstitute.org/mammals/haploreg/detail_v2.php?query=&id=rs5914778) **(**Query SNP: rs5914778 and variants with r^2^ ≧ 0.8)

| **Chr** | **Pos (hg19)** | **LD** | **LD** | **Variant** | **Ref** | **Alt** | **AFR** | **AMR** | **ASN** | **EUR** | **Enhancer** | **Dnase** | **GENCODE** | **RefSeq** | **dbSNP** |
| --- | --- | --- | --- | --- | --- | --- | --- | --- | --- | --- | --- | --- | --- | --- | --- |
|  |  | **(r²)** | **(D')** |  |  |  | **freq** | **freq** | **freq** | **freq** | **histone marks** |  | **genes** | **genes** | **func annot** |
| X | 56758231 | 1 | 1 | [rs5914778](http://www.broadinstitute.org/mammals/haploreg/detail_v2.php?query=&id=rs5914778) | A | G | 0.69 | 0.72 | 0.69 | 0.77 | Huvec, HSMM | BLD (GM12878_Lymphoblastoid) | RP11-622K12.1 | *LINC01420* | intronic |

Annotation of rs5914778 from HaploReg v2

LD, Linkage disequilibrium;
