# Supplementary material for: Discovery of a novel genetic susceptibility locus on X chromosome for systemic lupus erythematosus
Source: Arthritis Res Ther. 2015 Dec 3;17:349. doi: 10.1186/s13075-015-0857-1 (PMC4669597; doi:10.1186/s13075-015-0857-1)
Supplement: Additional file 3: Table S3. — Presenting the motifs predicted to be affected by rs5913992 SNP (Regulome DB). (DOC 171 kb) [file 13075_2015_857_MOESM3_ESM.doc]

**Additional file 3: Table S3. The motifs predicted to be affected by rs5913992 SNP (Regulome DB)**

| **Method** | **Location** | **Motif** | **Cell Type** | **PWM** | **Reference** |
| --- | --- | --- | --- | --- | --- |
| Footprinting | chrX:56759986..56760004 | EWSR1-FLI1 | Huvec | 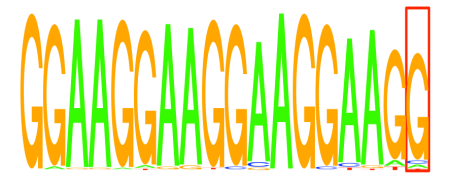 | 21106904 |
| Footprinting | chrX:56759986..56760004 | EWSR1-FLI1 | Mcf7 | 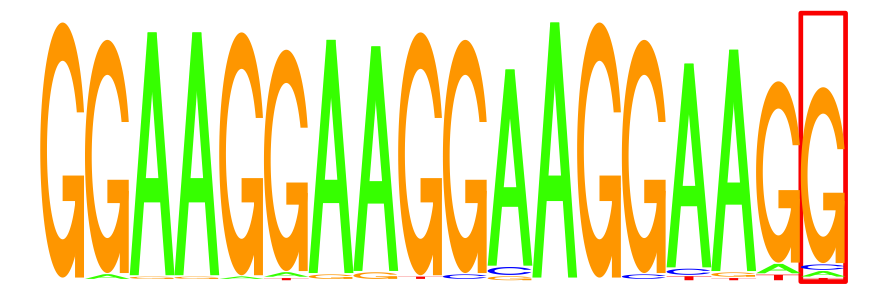 | 21106904 |

The consensus sequence logo of transcription factor binding motifs EWSR1-FLI1 with the red bar indicating the target position of rs5913992.
